# Approach and overview of autoimmune encephalitis: A review

**DOI:** 10.1097/MD.0000000000042472

**Published:** 2025-05-23

**Authors:** Yavuz Yucel, Nor Osman Sidow, Ahmet Yilmaz

**Affiliations:** a Department of Neurology, Dicle University Faculty of Medicine, Diyarbakir, Turkey; b Department of Neurology, Mogadishu Somalia-Türkiye Training and Research Hospital, Mogadishu, Somalia; c Department of Family Medicine, Dicle University Faculty of Medicine, Diyarbakir, Turkey.

**Keywords:** autoantibody, autoimmune encephalitis, epilepsy, immunotherapy, limbic

## Abstract

Autoimmune encephalitis (AE) is an inflammatory disease of the brain parenchyma that is mediated by many specific autoantibodies and is not caused by bacterial or viral causes. A diverse and growing number of autoantibodies have been identified in association with different types of AE. These antibodies can target either intracellular or cell-surface antigens. Advances are being made in understanding the clinical spectrum and treatment of AE. The prevalence and incidence of AE are increasing, although they remain comparable to those of infectious etiologies. The clinical presentation and management of AE are complex, with overlapping features. AE should be considered in the differential diagnosis of treatment-resistant epilepsy. Prompt diagnosis and treatment are critical in preventing seizures from developing into epilepsy. However, the broad differential diagnosis, the inability to detect specific autoantibodies in every patient, and delays in receiving antibody test results impede early diagnosis and treatment. Immunosuppressive agents are primarily used in treatment; first-line options include corticosteroids, intravenous immunoglobulin, and plasmapheresis, while rituximab and cyclophosphamide are used as second-line treatments. This review aims to provide a concise and accessible summary of this topic for readers and researchers.

## 1. Introduction

The term “encephalitis” is defined as inflammation of the brain parenchyma associated with neurological dysfunction (e.g., altered mental status, behavior, or personality change; motor or sensory deficits; speech or movement disorders; and seizure) for the presence of central nervous system inflammation. Central nervous system (CNS) inflammations are classified according to the site of inflammation: encephalitis, which refers to inflammation of the brain parenchyma, and meningitis, which refers to inflammation of the meninges.^[1]^

The estimated annual incidence of encephalitis worldwide is 7 to 15 cases per 100,000 patients. It has been repeatedly demonstrated that people over 65 are more likely than younger people to get encephalitis and other CNS diseases, as well as to experience the negative consequences that come with them.^[1]^

Encephalitis can be caused by infections or autoimmune diseases. Clinical, laboratory, neuroimaging, and electrophysiological results are commonly used to make a diagnosis. Autoimmune encephalitis (AE) affects the brain parenchyma, including the white matter, meninges, and spinal cord, as well as cortical and deep gray matter.^[2]^ Epidemiological studies suggest that the annual incidence of AE is 8 to 15 cases per 1,000,000 people. Anti N-methyl D-aspartate receptor encephalitis, first identified in 2005, has a female-to-male ratio of 4:1 and a mean age of onset of 21 years.^[3]^ It is important to obtain a detailed history, including travel and exposure to vectors or any other acute illness.^[4]^ All patients presenting with suspicion of encephalitis should have a lumbar puncture, blood cultures, and human immunodeficiency virus should be checked.^[1,4]^

The discovery of autoantibodies has been a turning point in AE research. The number of autoantibody tests detected has been increasing year by year and the number of laboratories has also increased. Thanks to these developments, AE diagnosis is faster and earlier than in the past. This narrative review of related articles is not the first review of AE, but it provides a concise, easy-to-read, and comprehensive summary of the full background of AE.

## 2. Method

A comprehensive literature search was conducted using PubMed and Google Scholar to explore to understand the features of AE such as diagnosis, classification, etiopathogenesis, historical development, types, follow-up features. Selected articles were reviewed and additional relevant studies were identified from references. Data were extracted and synthesized to provide an overview of traditional and innovative management strategies, including the application of advanced with biochemical parameters in AE identification features care and prognosis. A total of 251 articles were recovered. Every article underwent a rigorous evaluation, and its quality was ranked according to the degree of evidence it contained, which is determined by particular scientific techniques that influence the reliability of a study’s findings. No ethical approval or patient consent was required because all narration and content were based on previous literature review.

### 2.1. Overview of AE

AE is a group of syndromes with paraneoplastic or immunological etiology, usually characterized by subacute onset of memory impairment, confusion, and frequent seizures.^[2,4]^ An increasing number of types of AE is being identified across a broad clinical spectrum, ranging from epilepsy to movement disorders to psychosis. Autoimmune (immune = mediated) encephalitis includes diseases related to antibodies against neuronal cell-surface synaptic proteins as well as those linked with antibodies against intracellular neuronal proteins.^[5]^ Although both types of AE can present as paraneoplastic symptoms of an underlying cancer, the prevalence of cancer connection differs according to the autoantibodies. Most neural antibodies associated with paraneoplastic encephalitis (e.g., >70 percent of patients have a cancer association) target intracellular neuronal proteins (onconeuronal proteins).^[6]^ The discovery of new antibodies and associated disorders has led to the development of autoimmune neurology, a rapidly evolving discipline.^[2,7]^

AE is a challenging clinical diagnosis since numerous kinds of autoimmune and infectious encephalitis have comparable clinical, imaging, and laboratory findings. Patients usually come to the doctor with a memory impairment lasting days or weeks. Anamnesis and neurological examination may provide some or no clues.^[8]^

### 2.2. Historical epidemiological characteristics of AE

Since 1968, limbic encephalitis has been recognized as a rare paraneoplastic syndrome. Antineuronal or paraneoplastic antibodies (such as antiHu, Ma2/Ta, collapsin response mediator protein 5, and amphyphisin) were originally found in the 1980s and 1990s.^[9]^ In 1995, it was discovered that some individuals with neuromyotonia developed antibodies against the voltage gated potassium channel (VGKC) and responded to immunotherapy through plasma Exchange.^[10]^ In 2010, 2 large research groups found that most individuals with anti-VGKC encephalitis had antibodies that bound to leucine-rich glioma-inactivated protein 1 (LGI1).^[11]^

The incidence of encephalitis among adults in Western countries ranges from 0.7 to 12.6 per 100.000 people.^[12]^ Infections causes the majority of encephalitis cases.^[13]^ However, patients with antibodies to neuronal cell-surface proteins are increasingly being identified in research and clinical settings, suggesting an autoimmune etiology.^[13]^ According to a recent Olmsted County epidemiological study, the frequency of AE (0.8 per 100,000) is similar to that of infective encephalitis (1 per 100,000).^[14]^

### 2.3. Pathophysiology of AE

AE exhibits diverse pathophysiological and clinical subgroups. The first category includes classic paraneoplastic illnesses caused by intracellular antigen antibodies, such as anti-Hu.^[15]^ These conditions are strongly associated with cancer and are characterized by T-cell responses that target neurons. The second category includes autoantibodies against extracellular epitopes of channels, receptors, and other proteins, such as the anti-N-methyl-D-aspartate receptor.^[16]^ Diseases characterized by autoantibodies to intracellular synaptic proteins, such as antibodies against glutamic acid decarboxylase 65 (GAD65), fall into an intermediate category; it remains unclear as this does not fit neatly into either of these defined categories.^[17]^ Other types of AE lacking specific antigens, such as lupus cerebritis or acute disseminated encephalomyelitis, are rare neurological conditions that usually occur after exposure to a viral, bacterial, or autoimmune agent. They cause inflammation in the central nervous system, with common symptoms including headache, confusion, weakness, and paresthesia in the last group of acute disseminated encephalomyelitis.^[18]^

### 2.4. Clinical features

Some antibody-mediated syndromes of AE have specific clinical symptoms; however, these may appear in only a small percentage of patients at an early or late stage of the illness, when therapy should have already been initiated. In some situations, individuals may present a phenotype that overlaps with multiple antibody-mediated encephalitides, without specific pathognomonic symptoms.^[19]^

### 2.5. Diagnostic criteria for possible AE

Diagnosis can be made when all 3 of the following criteria have been met:

1. Subacute onset (rapid progression of <3 months) of working memory deficits (short-term memory loss), altered mental status, or psychiatric symptoms
2. At least one of the following:

New focal central nervous system findings;
Seizures not explained by a previously known seizure disorder;
Cerebrospinal fluid pleocytosis (white blood cell count of more than 5 cells per mm^3^);
Magnetic resonance imaging (MRI) features suggestive of encephalitis.

3. Reasonable exclusion of alternative causes

AE typically manifests as a subacute (days to a few weeks) progressive reduction in consciousness, often accompanied by fluctuations or a decrease in cognitive function.^[2,20]^ Early in the course of the disease, memory (especially the ability to retain new knowledge) may be compromised. Psychiatric symptoms are also frequently observed early on and can include psychosis, aggression, terror, panic attacks, compulsive behaviors, and inappropriate sexual conduct.^[21]^ Additionally, movement disorders and seizures are common in AE. A distinct form of cerebellitis causes ataxia and gait disorders, with nystagmus and vertigo frequently seen in AE.^[8,22]^ The differentiation between autoimmune and infectious encephalitis is shown in Table 1.

**Table 1:** Clinical features that differentiate autoimmune from infectious encephalitis.

Feature	Autoimmune encephalitis	İnfective encephalitis
Fever	‐	+
Systemic response	‐	+
İnfective prodromal	±	+
Temporal onset	Days to weeks	Hours to weeks
Course	Detorioration with fluctuations	Deterioration with fluctuations
Frequency of seizures	High frequency from onset	Variable
Cerebrospinal fluid white cell count	Usually < 100	Usually 100s–1000s
Cerebrospinal fluid protein	Mild elevation	Mild to high elevation
Cerebrospinal fluid oligoclonal bands	Negative paired Unpaired polyclonal	Negative paired Unpaired polyclonal
Magnetic resonance imaging brain	Often normal Focal inflammatory lesions enhancement	Often significant abnormalities

### 2.6. Autoimmune limbic encephalitis

The clinical features of limbic encephalitis include mood swing, working memory impairment, disorientation that develops quickly, often accompanied by seizures. The subacute onset of short-term memory loss is considered the defining feature of this disorder. Since immune-mediated limbic encephalitis can develop without detectable antibodies, the presence of antibodies is not required to confirm a definitive autoimmune origin.^[23]^ Cerebrospinal fluid (CSF) investigations typically reveal mild to moderate lymphocytic pleocytosis. Nonetheless, measuring autoantibodies remains crucial, as it helps identify the immunological subgroup of limbic encephalitis, which can vary in prognosis, tumor associations, and comorbidities depending on the specific autoantibody.^[19,24]^

### 2.7. The diagnostic criteria for definitive of limbic encephalitis

Diagnosis can be made when all 4 of the following criteria are met:

1. Subacute onset (within 3 months) of working memory deficits, seizures, or mental symptoms indicating limbic system volume.
2. MRI brain which shows bilateral abnormalities that were mostly limited to the medial temporal lobes on T2-weighted flair.
3. One or more of the followings:

Cerebrospinal fluid pleocytosis (more than 5 white blood cells per min^3^)
Electroencephalography (EEG) showing slow-wave or epileptic activity in the temporal lobes.

4. Reasonable exclusion of alternative causes.

**Figure 1.**
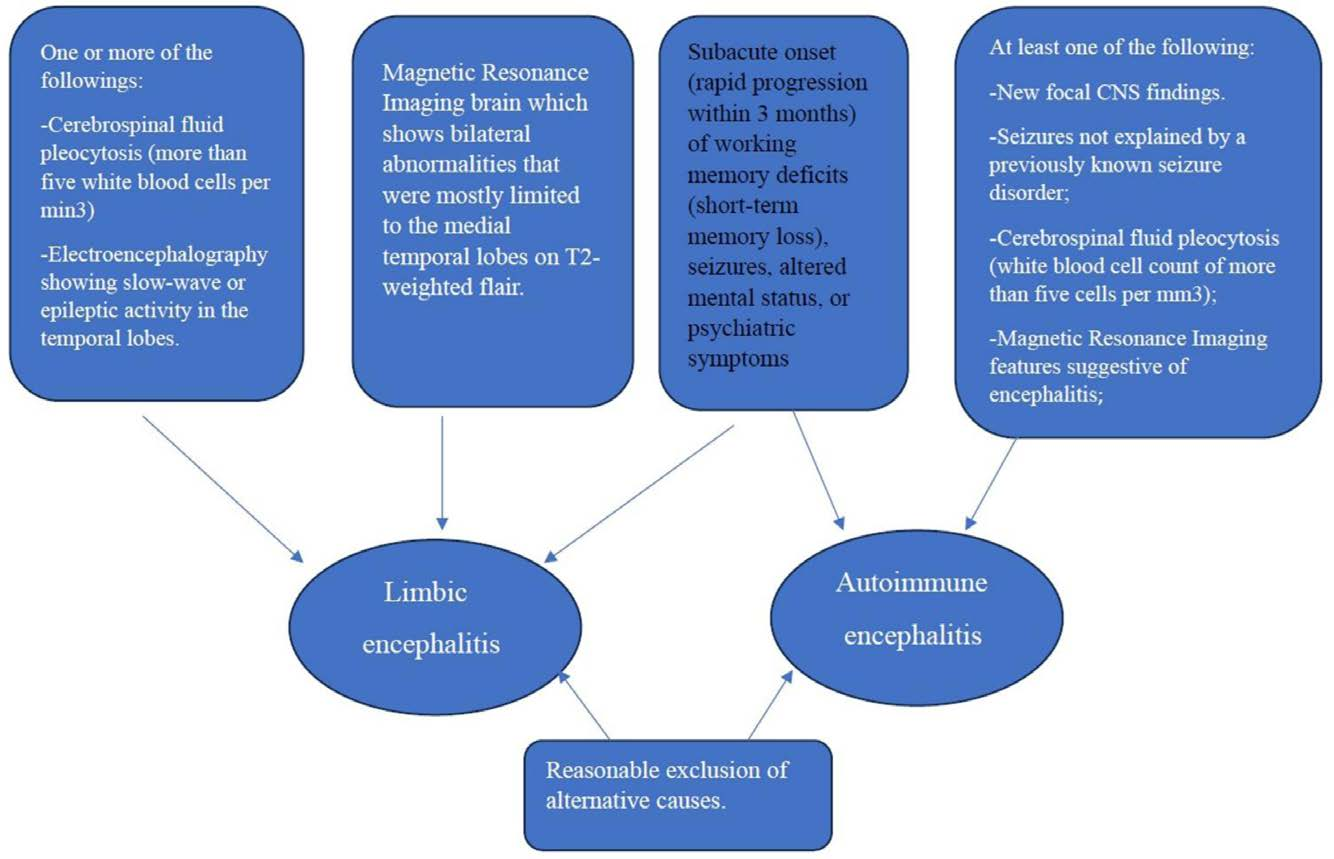
Diagnostic criteria for autoimmune encephalopathy and limbic encephalitis and their interactions.

Diagnostic criteria for autoimmune encephalopathy and limbic encephalitis and their interactions Fig. 1.

### 2.8. Anti-N-methyl-D-aspartate receptor encephalitis

The N-methyl-D-aspartate (NMDA) receptor is the main excitatory receptor that binds glutamate, the brain’s primary neurotransmitter, and is present in almost every brain region. Therefore, clinical symptoms are highly diverse. Anti-N-Methyl-D-aspartate receptor encephalitis is a distinctive neuropsychiatric disease most common in young women and children, although it can also affect men and elderly patients of both sexes. It is the most commonly identified cause of AE.^[25]^ Adults typically present with complex psychopathology, such as psychosis, delusions, hallucinations, agitation, or catatonia. In younger patients (under 18 years), the earliest symptoms are typically neurological (e.g., dyskinesias, seizures).^[26]^ Within days to a few weeks of presentation, all patients develop autonomic and respiratory instability, necessitating intensive care support. Over half of these patients have an accompanying tumor, most commonly an ovarian teratoma. The CSF usually exhibits lymphocytic pleocytosis, albeit less frequently.^[27]^ Early immunotherapy is correlated with better outcomes in NMDA receptor encephalitis patients; second-line therapy should be initiated if first-line medications fail. The anti-NMDA receptor encephalitis one-year functional status score, derived from 328 patients, is an early clinical tool for predicting prognosis.^[28]^

### 2.9. Anti-gamma-aminobutyric acid^B^ receptor encephalitis

Anti-gamma-aminobutyric acid^B^ receptor encephalitis is the third most frequently identified class of antineuronal cell–surface protein antibodies and likely targets the GABA^B^ receptor in both males and females; approximately half of the cases are associated with a tumor, often a lung neuroendocrine tumor or small-cell lung cancer, and are primarily characterized by very frequent, treatment-resistant focal seizures and mild mental and cognitive symptoms.^[29,30]^

### 2.10. Anti-leucine-rich glioma inactivated 1 limbic encephalitis

Anti-leucine-rich glioma-inactivated 1 limbic encephalitis primarily affects older patients who typically present with the classic form of limbic encephalitis. It generally manifests in 1 of 2 ways: rapidly progressing cognitive decline or new-onset, high-frequency, focal seizures.^[31]^ Patients with cognitive dysfunction frequently exhibit hyponatremia due to inappropriate antidiuretic hormone secretion patterns. Some patients may experience ictal arrhythmias, ranging from benign electrocardiogram changes to complete heart block. While the CSF is usually normal, there may be mild inflammatory changes or oligoclonal bands. Although antibodies are often detected in the CSF, intrathecal antibody production is not typically involved for unknown reasons. The MRI results are similar to those seen in other types of limbic encephalitis.^[32]^

### 2.11. Anti-gamma-aminobutyric acid^A^ receptor encephalitis

Anti-gamma-aminobutyric acid^A^ receptor encephalitis primarily affects children and adults between 3 and 63 years old, with rapidly progressing encephalopathy, refractory seizures, and epilepsy partialis continua. Patients with this syndrome rarely have an underlying tumor, but when present, it is typically associated with thymoma. Some patients have unrelated autoantibodies (e.g., thyroid peroxidase and GAD65).^[33]^ The CSF typically shows lymphocytic pleocytosis, elevated protein content, and occasionally oligoclonal bands. MRI findings in these patients exhibit multifocal and extensive flair T2 abnormalities. Immunotherapy appears to be effective in treating this form of encephalitis.^[34]^

### 2.12. Anti-alpha-amino-3-hydroxy-5-methyl-4-isoxazolepropionic acid receptor encephalitis

Anti-alpha-amino-3-hydroxy-5-methyl-4-isoxazolepropionic acid (AMPA) receptor encephalitis is characterized by the clinical features of limbic encephalitis, such as subacute onset of confusion, disorientation, and memory loss, which are commonly seen in this disorder. Approximately 70% of affected individuals are middle-aged women, and the condition is often paraneoplastic.^[35]^ Cancers frequently linked to this condition include thymus, lung, and breast cancers. CSF results are comparable to those found in anti-NMDA receptor encephalitis, with predominant lymphocytic pleocytosis. Brain MRI typically shows an abnormal flair signal in the medial temporal lobes.^[15,34,36]^

### 2.13. Anti-contactin-associated protein like 2 associated encephalitis

Anti-contactin-associated protein like 2 (CASPR2) associated encephalitis typically presents in patients with symptoms of limbic encephalitis, peripheral nerve hyperexcitability or neuromyotonia, and Morvan syndrome; the vast majority of patients do not have antibodies. Some patients also have antiacetylcholine or muscle-specific kinase antibodies alongside other immune-mediated diseases.^[37]^ CASPR2 affects the juxtaparanodal area of myelinated axons, as shown in brain MRI. Abnormalities in this gene are linked to refractory seizures, schizophrenia, psychosis, and autism. Making the right diagnosis is essential, as immunotherapy for anti-CASPR2-related encephalitis has been shown to improve outcomes in mental function and, in certain cases, neuromyotonia.^[38]^

### 2.14. Anti-dipeptidyl-peptidase-like protein-6 encephalitis

Anti-dipeptidyl-peptidase-like protein-6 (DPPX) encephalitis was first observed in 4 adults aged 45 to 76 years who experienced agitation, hallucinations, myoclonus, seizures, and rapidly progressing encephalopathy. The illness may be severe enough to require extended hospital stays. DPPX is expressed by neurons in the myenteric plexus and is important for regulating the Kv4.2 ion channel VGKC, although no correlation exists between this condition and cancer. Severe diarrhea and weight loss often raise the suspicion of a paraneoplastic cause of the disorder.^[39]^

### 2.15. Other autoimmune encephalitis

Amphiphysin-related antibodies are associated with paraneoplastic stiff person syndrome. GAD65 are rarely seen in cancer-related cases, although they are frequently related to nonparaneoplastic stiff person syndrome.^[40]^ Some patients with idiopathic or paraneoplastic cerebellar ataxia linked to Hodgkin lymphoma have antibodies against metabotropic glutamate receptor 1.^[41]^ Dopamine-2 receptor antibodies have been observed in a subset of individuals with Tourette syndrome, as well as in children with Sydenham chorea and basal ganglia encephalitis, although none of these conditions are associated with malignancy.^[16,17]^ The clinical syndromes of AE and its autoantibodies are shown in Table 2.

**Table 2:** Syndromes of autoimmune encephalitis linked to antibodies directed against synaptic antigens and the surface of neurons.

Antigen	Clinical features	Age range (median) in years	Sex-ratio (F:M)	Tumor associations and frequency
NMDA receptor	Prodromal syndrome, psychiatric manifestations, seizures, amnesia, movement disorder, autonomic instability	0.6 = 85(21)	4:1	Age dependent ovarian teratoma 10 = 50%
LGI1	Limbic encephalitis, seizures	30 = 80 (60)	1:2	Thymoma, thyroid, lung, and renal cell reported <10%of all tumors combined
CASPR2	Morvan syndrome, neuromyotomnia, limbic encephalitis	46 = 76 (60)	1:4	Thymoma 0 = 40%
AMPA receptor	Limbic encephalitis, psychiatric syndromes	38 = 87 (60)	9:1	Lung, breast, thymoma, thymic carcinoma Up to 70% for all tumors combined
GABA^B^ receptor	Limbic encephalitis, seizure	16 = 77 (62)	2:3	Small cell lung cancer 50% more seen in elder patients
GABA^A^ receptor	Refractory seizures, status epilepticus	3 = 63 (22)	1:510 = 50%	Thymoma in one of the seizure cases
DPPX	Encephalitis, CNS hyperexcitability, agitation, hyperekplexia, movement disorders seizures, progressive encephalomyelitis with rigidity and myoclonus	45 = 76	1:2	No tumors was described

AMPA = anti-alpha-amino 3-hydroxy 5-methyl 4-isoxazolepropionic acid, gamma aminobutyric acid, CASPR2 = anticontactin associated protein like 2, CNS = central nervous system, DPPX = anti-dipeptidyl peptidase like protein-6, LGI1 = leucine rich glioma inactivated protein 1, NMDA = N-methyl D-aspartate.

### 2.16. Diagnostic approach

The primary diagnostic tools used to confirm a diagnosis of AE include antibody testing, imaging, EEG, and biopsy with cancer screening.

### 2.17. Antibody testing

For AE to be properly diagnosed, autoantibody testing is crucial. However, these tests are complex and must be interpreted carefully; it can be erroneous to consider some test results as definitive proof of AE. Many commercial tests are available for autoantibodies to the NMDA, LGI1, CASPR2, AMPA receptor, and GABA^B^ receptors.^[42]^ Clinical testing for more recent cell surface antigens, such as DPPX and the GABA^A^ receptor, is more challenging.^[43]^ Both the traditional intracellular neuronal antibodies and the synaptic intracellular antigens GAD65 and amphiphysin are readily accessible. Cerebrospinal fluid testing for NMDA and other surface antibodies is the most specific and sensitive. Serum testing can yield a low false positive rate but also a high false negative rate.^[43]^ Examples of IgG responses are pathogenic cell-surface or synaptic autoantibodies, including CASPR2 and LGI1.^[44]^

### 2.18. Imaging

**Figure 2.**
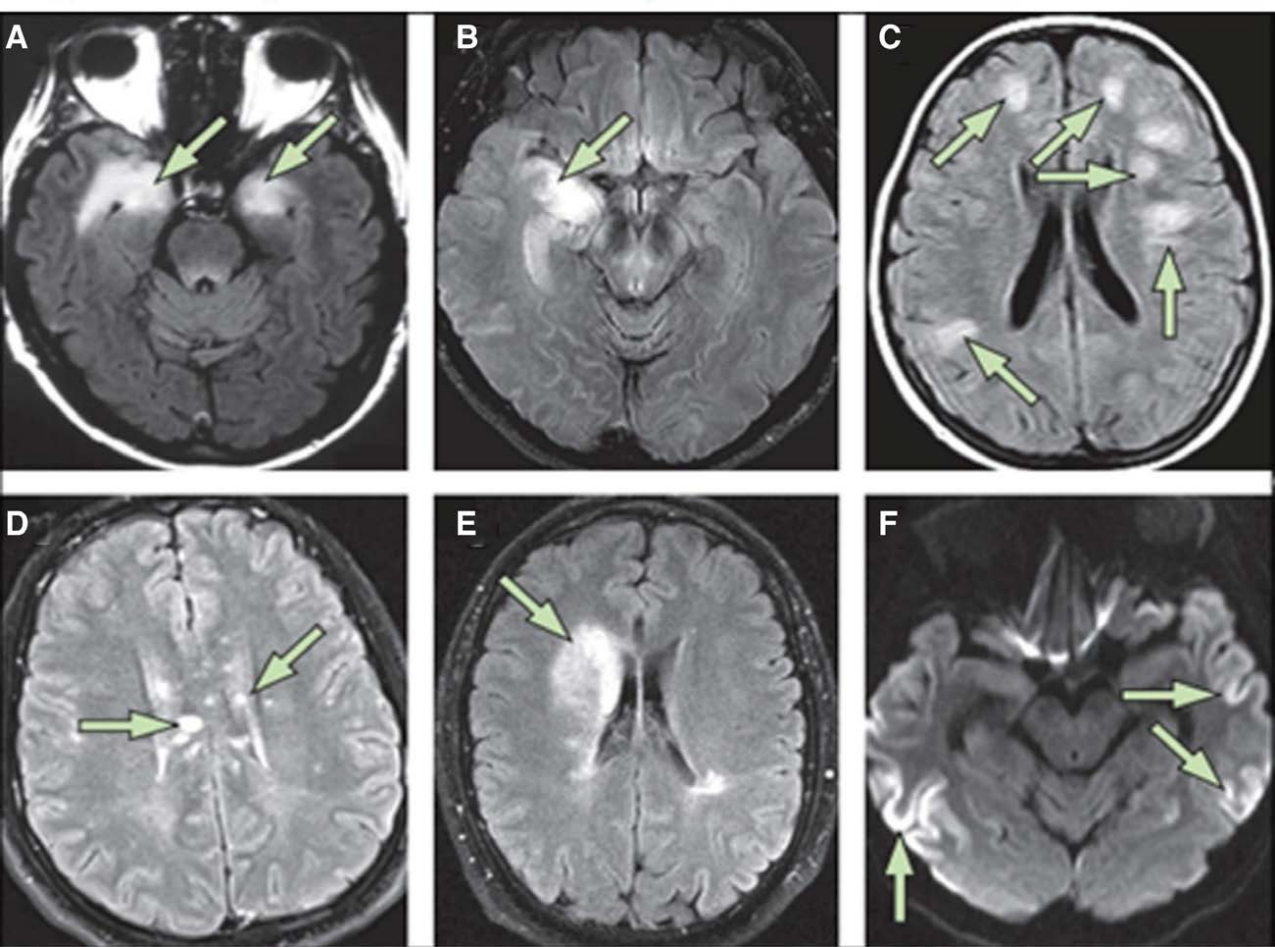
MRI sequences in autoimmune encephalitis and its mimics. Standard limbic encephalitis MRI (A) showing bilateral abnormalities in the medial temporal lobe on T2-weighted fluid-attenuated inversion recovery imaging; antineuronal antibodies were not detected in the serum or CSF of this autopsy-confirmed limbic encephalitis patient. The patient had a final diagnosis of glioblastoma (B) and showed signs of limbic encephalitis-like unilateral right hippocampus involvement. An acute disseminated encephalomyelitis (C) typical MRI shows bilateral massive lesions in the white matter. A patient suffering from Susac syndrome (D) has several lesions impacting the corpus callosum. An MRI of a patient with overlapping conditions (anti-NMDA receptor and myelin oligodendrocyte glycoprotein antibodies (E) reveals a demyelinating abnormality that is compatible with a right frontal. An MRI sequence that resembles the alterations observed in individuals with Creutzfeldt-Jakob disease is observed in a patient suffering from AMPA receptor antibody-associated encephalitis (F). AMPA = anti-alpha-amino 3-hydroxy 5-methyl 4-isoxazolepropionic acid, CSF = cerebrospinal fluid, MRI = magnetic resonance imaging, NMDA = N-methyl-D-aspartate.

In patients with NMDA, LGI1, CASPR2, or other autoantibodies, advanced imaging studies such as positron emission tomography or single photon emission computed tomography have detected metabolic changes in certain brain regions. However, these research methods are not sufficient for definitive and differential diagnosis. Patients with GABA^B^, LGI1, CASPR2, NMDA, and AMPA receptor antibodies may have normal brain MRIs or may show elevated T2 signals, particularly in the medial temporal lobes.^[45]^ This pattern resembles that of other viral forms of encephalitis, such as herpes simplex virus encephalitis, in which 96% of patients display abnormalities on MRI. Other disorders, such as syphilis or tuberculosis, may manifest similarly. Less typical results may be associated with autoantibodies to GABA^A^ or DPPX. Therefore, brain MRI alone cannot differentiate between autoimmune and infectious causes.^[22,46]^ MRI images of AE are shown in Fig. 2.

### 2.19. Electroencephalography

EEG is useful for detecting subclinical epileptic seizures in patients with encephalitis, determining prognosis, and recommending symptomatic treatment. Normal EEG results are correlated with positive outcomes, regardless of other prognostic variables.^[47]^ The extreme delta brush pattern is seen in patients with anti-NMDA receptor encephalitis, most commonly in comatose patients. This distinctive EEG pattern should prompt testing for anti-NMDA receptor encephalitis antibodies. Patients with anti-NMDA receptor encephalitis and other types of AE may experience prolonged periods of unresponsiveness and unusual behavior not caused by seizure activity. Therefore, long-term EEG monitoring may be beneficial. Status epilepticus can arise in various types of AE,^[48]^ with the greatest risk appearing in individuals with autoantibodies targeting the brain’s primary inhibitory pathways.

### 2.20. Biopsy and cancer screening

Biopsy results are rarely definitive in autoimmune cases. Overall, the clinical impact of a biopsy performed for suspected encephalitis is minimal, with only about 8% of cases showing clear benefits.^[49]^

Neuronal antibodies to intracellular antigens, such as Hu, are more frequently found in cancer patients than in autoimmune disease patients. For instance, in patients with small-cell lung cancer without anti-Hu neurological symptoms, a low-titer serum Hu response is a common diagnosis associated with ovarian teratoma, assessed using pelvic MRI or ultrasonography. Testicular ultrasonography is essential in young men with a diagnosis of Ma2 or suspected cases, while for women with anti-Yo antibodies, mammography or breast MRI, pap smears, and pelvic imaging may be most beneficial. Even in the absence of an identified specific antibody, patients with high-risk disorders, such as cerebellar degeneration, should undergo comprehensive screening. Screening is important, as tumors may be quite small when neurological symptoms first appear.^[42,44,50]^

### 2.21. Treatment approaches and challenges

Treatment possibilities for AE range from broad-spectrum immunosuppressants to targeted approaches that address antibody-mediated disease pathways. Corticosteroids, such as those used in most inflammatory diseases, are used to treat AE by broadly inhibiting the inflammatory process. However, corticosteroids have limited efficacy in AE treatment and can lead to systemic side effects due to their lack of specificity for antibody-mediated immune processes. Other treatments target specific stages in the progression of AE.^[2,8,22,23,44,51]^ These treatments focus on autoantibodies and immune mediators (intravenous immunoglobulin and plasma exchange), B cells and short-lived plasma cells (rituximab), and cytokines related to autoimmune and inflammatory processes (tocilizumab and low-dose interleukin-2). Antiproliferative drugs, such as cyclophosphamide, azathioprine, and mycophenolate mofetil, are used to reduce lymphocyte proliferation.^[24,52]^ Ongoing studies continue to investigate additional antibodies and specific immunotherapies targeting autoantibodies.^[53]^ The therapeutic agents most commonly used for AE are shown in Table 3.

**Table 3:** Therapeutic agents used for autoimmune encephalitis.

Treatment	Regimen
*First line immunotherapy* Methylprednisolone Intravenous immunoglobulin Plasma exchange	1 g daily, for 3 to 5 days 2 g/kg over 5 days (400mg/kg/day) 1 session ever other day for 5–7 cycles.
*Second line immunotherapy* Rituximab Cyclophosphamide Alternative therapy Tocilizumab Low dose interleukin (IL)-2 2 (aldesleukin)	375 mg/m^3^ weekly IV infusion for 1 week 750 mg/m^3^ monthly for 3 to 6 months · Initially 4 mg/kg, followed by an increase to 8mg/kg monthly based on clinical response. 1.5 million IU/day, 4 SC injections with 3 weeks interval
*Steroid sparing agents* Azathioprine Mycophenolate mofetil	Initially 1 to 1.5 mg/kg once daily or divided twice daily, target 2–3 mg/kg/day Initially 500 mg twice daily, target 1000 mg twice daily

### 2.22. Prognosis

Long-term functional outcomes are largely determined by the timing of immunotherapy initiation. Stabilization of neurological impairment is often regarded as a positive achievement because autoantibodies that target intracellular antigens typically do not respond, even in cases of severe immunosuppression.^[54]^ Patients with autoantibodies against cell–surface antigens generally have better outcomes. For individuals with paraneoplastic CNS disorders, the progression of the underlying malignancy is a primary cause of death.^[55]^

### 2.23. Limitations

This narrative review has several limitations that should be noted. First, the review relied exclusively on English-language articles and reports. This language restriction may have limited the inclusion of locally conducted studies or reports published in other languages, which might have provided unique regional insights, especially from areas where English is not the primary language. Second; according to previous data regarding AE is quite limited, so the literature is not enough presented for this topic and part of them is confused for the readers to summarize. Finally; the time frame for this part of review is still updating and recently appeared or attached by the researchers, so this topic needs to be reviewed or make it more wide range for the future researchers. it is essential to recognize these limitations and conduct comprehensive analysis and exploration within the field of AE.

## 3. Conclusion

Our recommendation as clinicians is that AE should be considered in the differential diagnosis of treatment-resistant epilepsy. Prompt diagnosis and treatment are critical in preventing seizures from progressing to epilepsy. AE is primarily diagnosed clinically, and treatment should begin early, without waiting for antibody test results. First-line treatments include steroids, intravenous immunoglobulin, and plasmapheresis, while rituximab and cyclophosphamide should be initiated as second-line options. If a tumor association is detected, surgery should be performed immediately. Most patients experience complete or significant recovery, although older age is correlated with a worse prognosis.

## Author contributions

**Conceptualization:** Yavuz Yücel, Nor Osman Sidow, Ahmet Yilmaz.

**Investigation:** Yavuz Yücel, Nor Osman Sidow, Ahmet Yilmaz.

**Methodology:** Yavuz Yücel, Nor Osman Sidow, Ahmet Yilmaz.

**Project administration:** Yavuz Yücel, Nor Osman Sidow, Ahmet Yilmaz.

**Resources:** Yavuz Yücel, Nor Osman Sidow, Ahmet Yilmaz.

**Writing – original draft:** Yavuz Yücel, Nor Osman Sidow, Ahmet Yilmaz.

**Writing – review & editing:** Yavuz Yücel, Nor Osman Sidow, Ahmet Yilmaz.
